# Epidemiological situation of Lyme borreliosis in Belgium, 2003 to 2012

**DOI:** 10.1186/s13690-015-0079-7

**Published:** 2015-07-03

**Authors:** Corinne Bleyenheuft, Tinne Lernout, Nicolas Berger, Javiera Rebolledo, Mathias Leroy, Annie Robert, Sophie Quoilin

**Affiliations:** WIV-ISP (Scientific Institute of Public Health), Rue Juliette Wytsmanstraat, 14, 1050 Brussels, Belgium; Department of Social and Environmental Health Research, London School of Hygiene and Tropical Medicine, 15-17 Tavistock Place, London, WC1H 9SH UK; Université catholique de Louvain - Brussels Campus, Institut de recherche expérimentale et clinique, Pôle de recherche Épidémiologie et Biostatistique, Public Health School -IREC-EPID B1.30.13, Clos chapelle aux champs 30, 1200 Brussels, Belgium

**Keywords:** Lyme borreliosis, Belgium, Epidemiology

## Abstract

**Background:**

Some studies show that the incidence of Lyme borreliosis is increasing in different European countries. In order to evaluate if this is also the case in Belgium, different data sources were consulted to describe the epidemiology of Lyme borreliosis in the country during the last decade.

**Methods:**

Data from two databases were analyzed for the time period 2003–2010 and 2003–2012 for respectively: the registration of minimal clinical data from Belgian hospitals (principal and secondary diagnosis), and a sentinel laboratory network reporting positive laboratory results.

**Results:**

The number of hospitalized cases per year remained stable between 2003 and 2010, ranging from 970 (in 2008) to 1453 (in 2006), with a median of 1132.5 cases per year. Between 2003 and 2012, yearly fluctuations in the number of positive tests were reported by the sentinel laboratory network (with a minimum of 996 positive tests in 2007 and a maximum of 1651 positive tests in 2005), but there is no increasing trend over the study period (median = 1200.5 positive tests per year). The highest incidence rates of hospitalization and the highest reported incidence of positive laboratory results are registered in the provinces of Luxemburg, Limburg, Flemish Brabant and Antwerp, with a typical seasonal pattern (peak in September). The age groups affected most are those from 5 to 14 years and 45 to 69.

**Conclusion:**

Based on hospital records and laboratory results, no increasing trend in Lyme disease was observed over the 2003–2012 period in Belgium. These results are in line with the stable incidence of erythema migrans reported by a sentinel network of general practitionners between 2003 and 2009. Multi-source surveillance of vector-borne diseases should be further implemented.

## Background

Lyme borreliosis is a multisystemic disease caused by spirochaetes belonging to the Borrelia burgdorferi sensu lato complex [1]. It is the most common tick-borne disease in North America and in Europe [2]. Historically, many syndromes, among which Bannwarth syndrome (painful radiculitis, cranial neuritis and lymphocytic meningitis) were reported since 1883 in Europe, and can retrospectively be designated as manifestations of Lyme borreliosis [2]. The spirochaete responsible for the disease was first discovered in ticks by W. Burgdorfer and collaborators in 1982, and afterwards named Borrelia burgdorferi sensu stricto [1]. It is a complex which comprises at least 19 genospecies [3, 4], and several of them are pathogenic to humans. The primary tick vector of the spirochaete in Europe is Ixodes ricinus [2, 3], and the main responsible of Borrelia transmission to humans are tick nymphs. They quest most actively from spring to autumn in microenvironments with more than 85 % relative humidity, such as deciduous or mixed woodland, as well as suburban and urban environments and roadsides [5].

The clinical manifestations in humans can be divided into three stages: early, early disseminated and late disseminated Lyme borreliosis [2, 6]. Early Lyme borreliosis (days to weeks) is most often characterized by a typical erythema migrans starting 3 to 30 days (typically after 7 to 14 days) after a tick bite. A borrelial lymphocytoma is rarely diagnosed, and described as a bluish red tumor-like skin infiltrate, often located at the earlobe or at the nipple. When the infection is untreated, the spirochete can disseminate and cause early disseminated Lyme borreliosis (weeks to months after the tick bite). Its manifestations include early neuroborreliosis, Lyme arthritis, multiple erythema migrans [7] or more seldom myocarditis with atrioventricular block. Finally, late manifestations of Lyme borreliosis can occur months to years after the infection, such as acrodermatitis chronica atrophicans, untreated Lyme arthritis, neuroborreliosis and possible autoimmune phenomena [2]. The vast majority of patients respond well to antibiotic treatment, with drug type, dose, route (oral or intravenous) and duration varying with the symptoms and the stage of the disease.

The diagnosis of Lyme borreliosis is mainly based on clinical symptoms (presence of erythema migrans, generally found in 60 to 80 % cases) and serological tests [2, 5]. Serological testing is recommended only 6 to 8 weeks after onset, and only in case of atypical, disseminated or late manifestations of the disease [2]. However, in the absence of clinical symptoms, the presence of anti-Borrelia antibodies does not necessarily indicate the presence of an active infection. Indeed, 4 to 20 % of the normal Western European population have detectable antibodies, most likely due to a (asymptomatic) Borrelia infection in the past. International guidelines recommend thus not to test for antibodies against Borrelia when the suspicion on Lyme borreliosis is low [2]. Furthermore, the duration of anti-Borrelia antibodies persistence in the human body is unknown. Reinfection in patients successfully treated by antibiotics has been described in the literature [8].

Some authors suggest that Lyme disease will become an important health concern in the coming years, especially in light of climate change predictions, which may impact on tick density [5]. Indeed, the annual number of Lyme borreliosis is increasing in some European countries (among others the Netherlands [9, 10], the United Kingdom [11], Hungary [12]), although not in others, like Germany [13], France [14] and Switzerland [15]. An increase in consultations and hospital admissions for Lyme borreliosis has also been described between 1994 and 2009 in the Netherlands, a neighboring country of Belgium [9, 10]. Concerns have thus arisen regarding the evolution of Lyme borreliosis in Belgium during the last decade, where some seroprevalence studies have been carried out in the past [1, 6, 16]. A study on the incidence of the disease based on data from a sentinel network of general practitioners did not show any increase of tick bite or erythema migrans incidence between the years 2003–2004 and 2008–2009 [17]. In the present study, we used routinely collected surveillance data to describe the epidemiology of Lyme disease in Belgium during the last ten years (2003–2012) and confirm or invalidate the stability described by the sentinel network of general practitioners [17].

## Methods

### Data collection

Two different data sources were used to collect data on Lyme borreliosis epidemiology in Belgium. Firstly, the number of hospitalizations, through the registration of minimal clinical data was collected to evaluate the burden of early disseminated and late manifestation of the disease. Secondly, data on positive results of laboratory tests reported by a sentinel network of laboratories were analyzed.

Since 1990, the Belgian Ministry of Health (federal public service Health, Food chain safety and Environment) collects compulsorily registered data (registration of minimal clinical data, RMC) from every general hospital in Belgium [18]. For each patient discharged, the physician has to fill in a standardized form summarizing medical records, and specifying all diagnosis. Data are then encoded following the International Classification of Diseases (ICD-9). In the ICD-9 classification, all Lyme borreliosis manifestations are grouped under the same code (088.81). We are thus unable to distinguish the type of complication leading to the hospitalization of the patient. At the time of this study, data were available until 2010. We therefore used RMC data (for all hospitalization wards) from 2003 to 2010, with Lyme borreliosis as principal and secondary diagnosis.

Since 1983, a sentinel laboratory network (SNL), coordinated by the Scientific Institute of Public Health (WIV-ISP) collects positive laboratory results on about 35 infectious diseases [19, 20], including Lyme borreliosis since 1987. The laboratories participate in this network on a voluntary basis. The absolute number of participating laboratories decreased over time due to fusions between laboratories, but the proportion of tests covered by the network remained globally stable. The network covers around 50 % of all laboratory tests carried out in Belgium (Berger N., unpublished observations). Each participating laboratory weekly reports the number of laboratory confirmed borrelia cases to the WIV-ISP, with information on test date, place of residence, gender and date of birth of the patient. A search for duplicates is performed within a calendar year, considering that a reinfection may occur after a one-year period.

The type of positive Lyme borreliosis test collected by the SLN changed over the study period (2003–2012). Indeed, a change in the NIHDI (National Institute for Health and Disability Insurance) reimbursement of serological tests occurred in 2008 in Belgium. Before 2008, only the ELISA screening benefited from a social security reimbursement. Since 2008, patients with a positive ELISA can benefit from a refund of the immunoblot assay to confirm ELISA screening. In our SLN database, data before 2008 contain thus the positive ELISA screening results. Since 2008, only positive blot assays (confirmation) are reported.

### Data analysis

RMC and SLN data were analyzed in terms of time, place and individual characteristics of the cases.

The geographical distribution was assessed by province, based on the address of the case, not the place of tick bite. For calculation of relative proportions (reported incidences), the number of cases was divided by the 2010 population of each province.

Reported cumulative incidences were compared at the provincial level throughout different time periods, using a non-parametric Wilcoxon test. RMC data were divided into two equal periods (2003–2006 and 2007–2010) to check the influence of a possible better knowledge of the disease at the end of the decade. The most recent period was then divided into two smaller time periods, and data were compared with each other. SLN data were divided into 2003–2007 and 2008–2012 periods, because of the laboratory test change, which occurred in 2008. Data from the most recent period were then compared between two smaller time periods.

All graphs and analyses were performed using SAS Enterprise Guide Software, version 5.1.

## Results

**Fig. 1:**
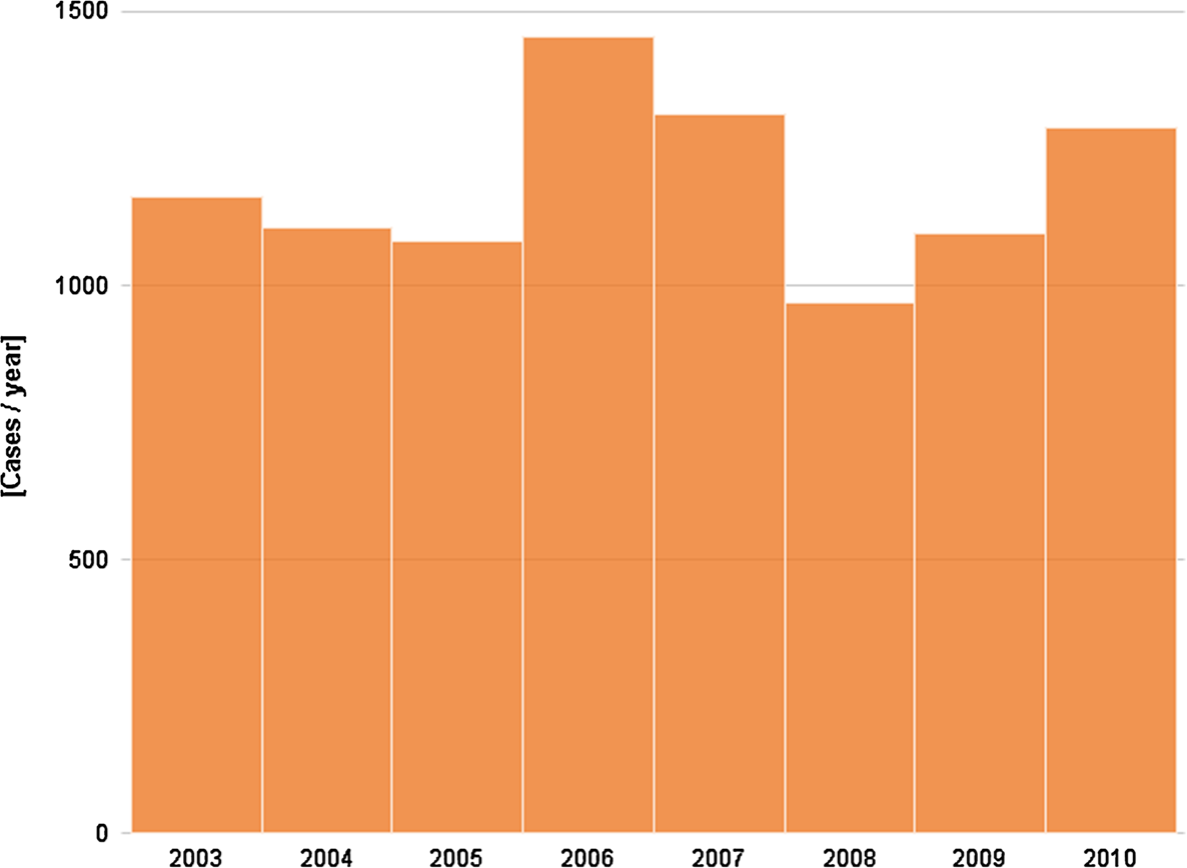
Number of hospitalizations for Lyme borreliosis (principal and secondary diagnosis) by year, 2003–2010, Belgium

**Fig. 2:**
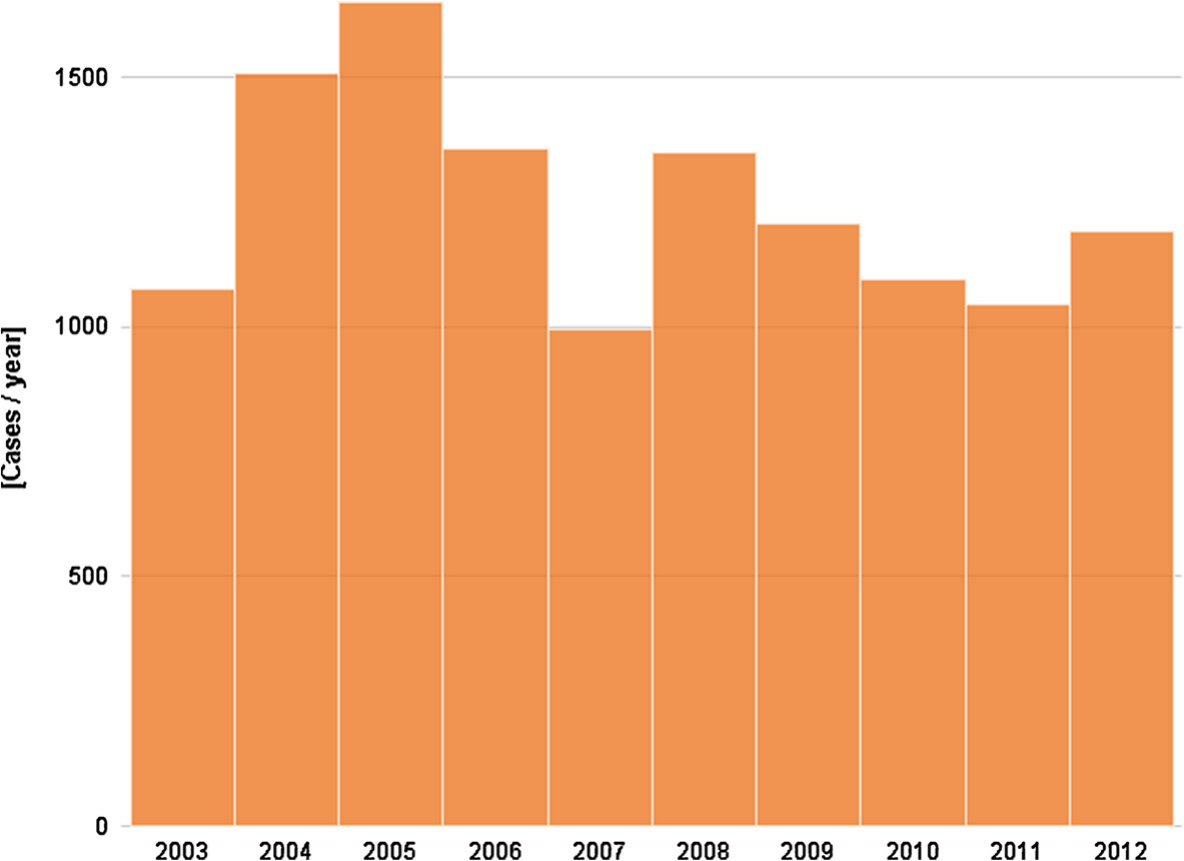
Number of Lyme borreliosis positive tests reported by the sentinel laboratory network, by year, 2003–2012, Belgium

Figures 1 and 2 present the number of hospitalizations per year between 2003 and 2010, and the number of positive tests per year between 2003 and 2012, respectively. No increasing trend is observed during the study period, with a number of hospitalizations ranging between a minimum of 970 in 2008 and a maximum of 1453 in 2006 (median = 1132.5 cases per year), and a number of SLN positive tests fluctuating between 996 in 2007 and 1651 in 2005 (median = 1200.5 positive tests per year).

**Fig. 3:**
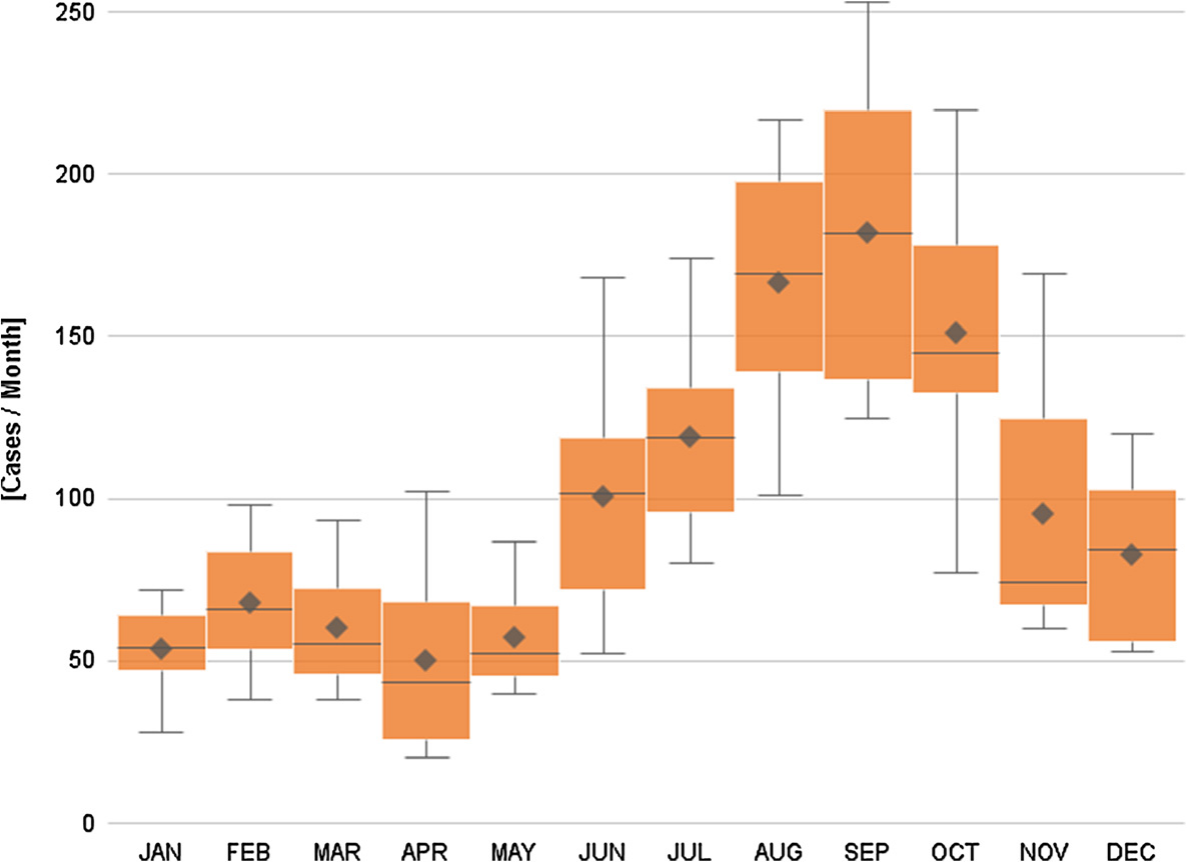
Number of hospitalizations for Lyme borreliosis, by month, 2003–2010, Belgium. Box plot: The mean and median annual number of cases are presented by month. The length of the box represents the interquartile range (the distance between the 25th and the 75th percentile). The horizontal line in the box represents the median, and the diamond, the mean. The wiskers extend to the group minimum and maximum value

**Fig. 4:**
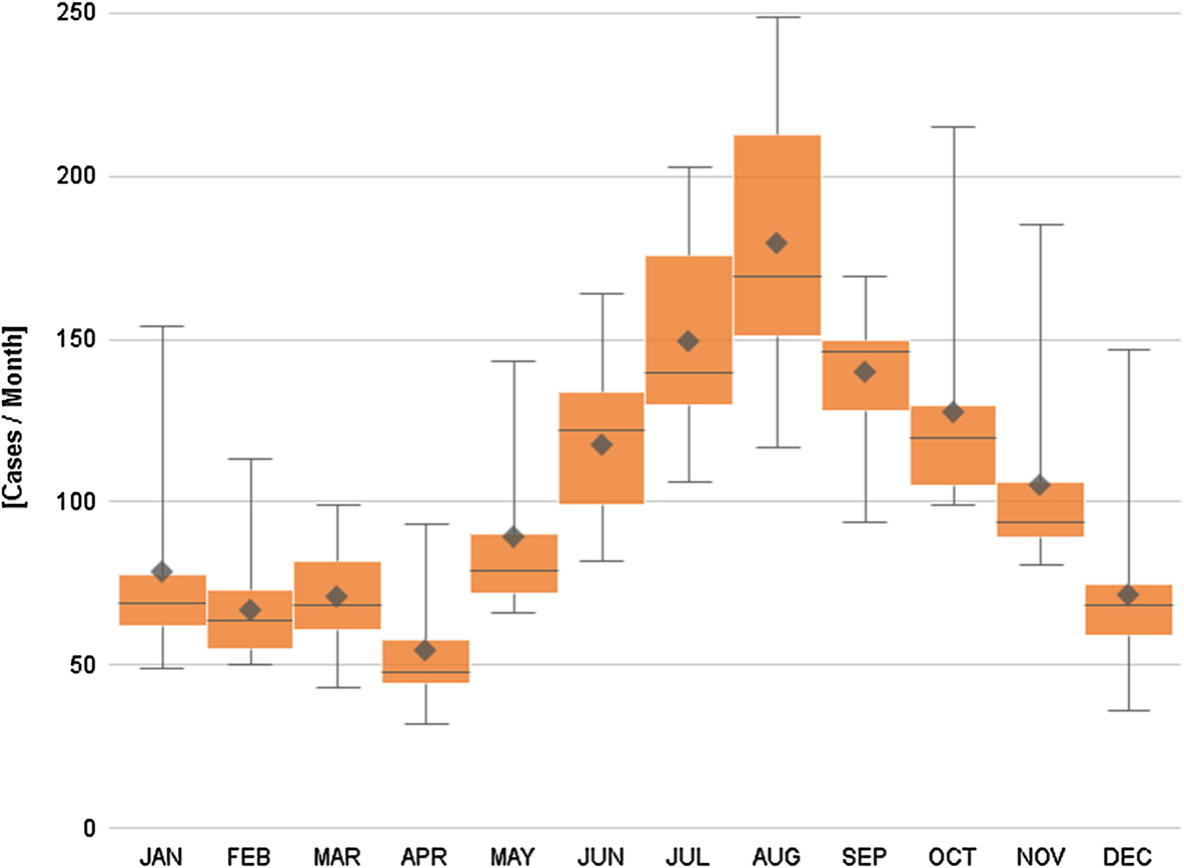
Number of positive laboratory tests for Lyme borreliosis, by month, 2003–2012, Belgium. Box plot: The mean and median annual number of positive tests are presented by month. The length of the box represents the interquartile range (the distance between the 25th and the 75th percentile). The horizontal line in the box represents the median, and the diamond, the mean. The wiskers extend to the group minimum and maximum value

The number of hospitalizations and the number of positive tests by month follow a seasonal pattern, with a minimal number of cases during the winter, a gradual increase during the spring and summer periods, and a maximum peak at the end of the summer and during the autumn (cumulative value for RMC: n = 1462 in September; SLN: n = 1794 in August), Figs. 3 and 4.

**Fig. 5:**
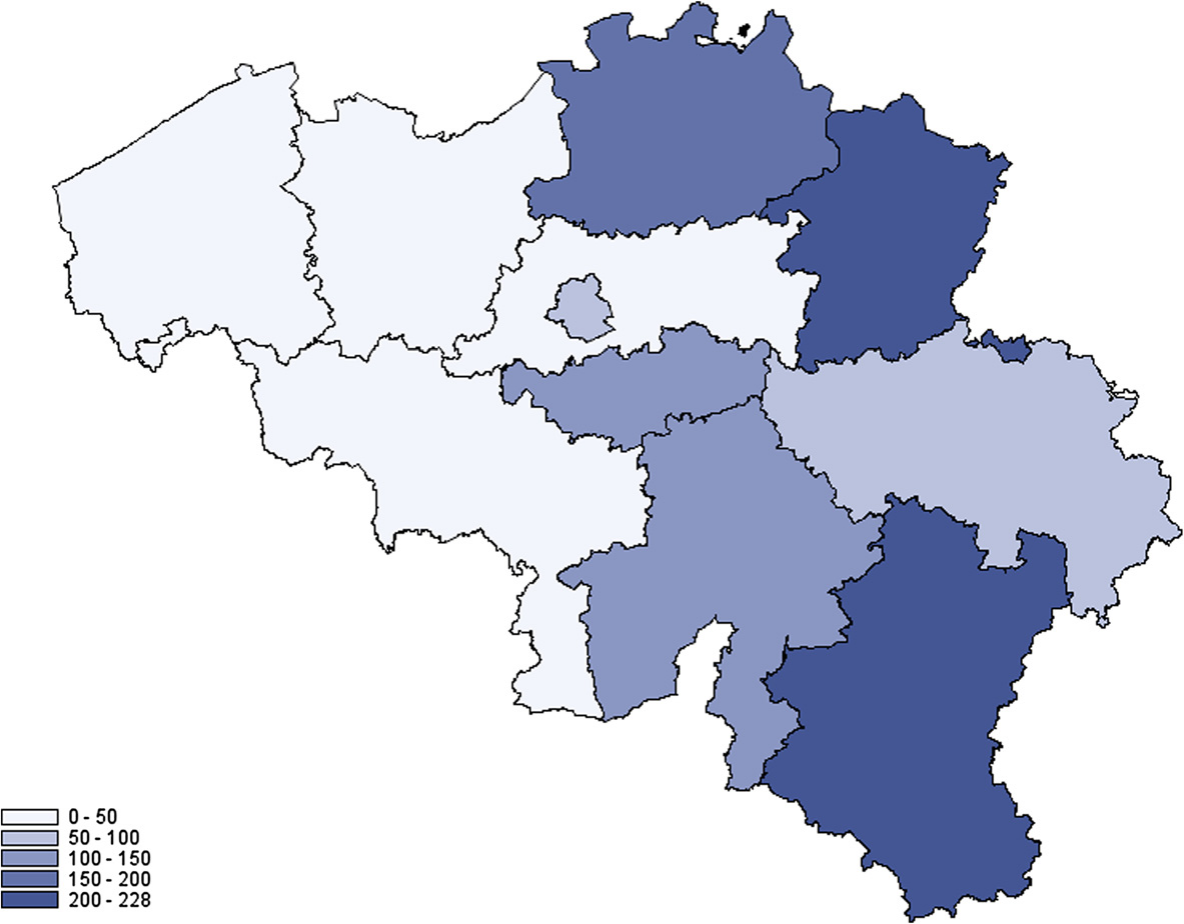
Cumulative incidence of hospitalizations for Lyme borreliosis by 100 000 inhabitants, by province, 2003–2010, Belgium

**Fig. 6:**
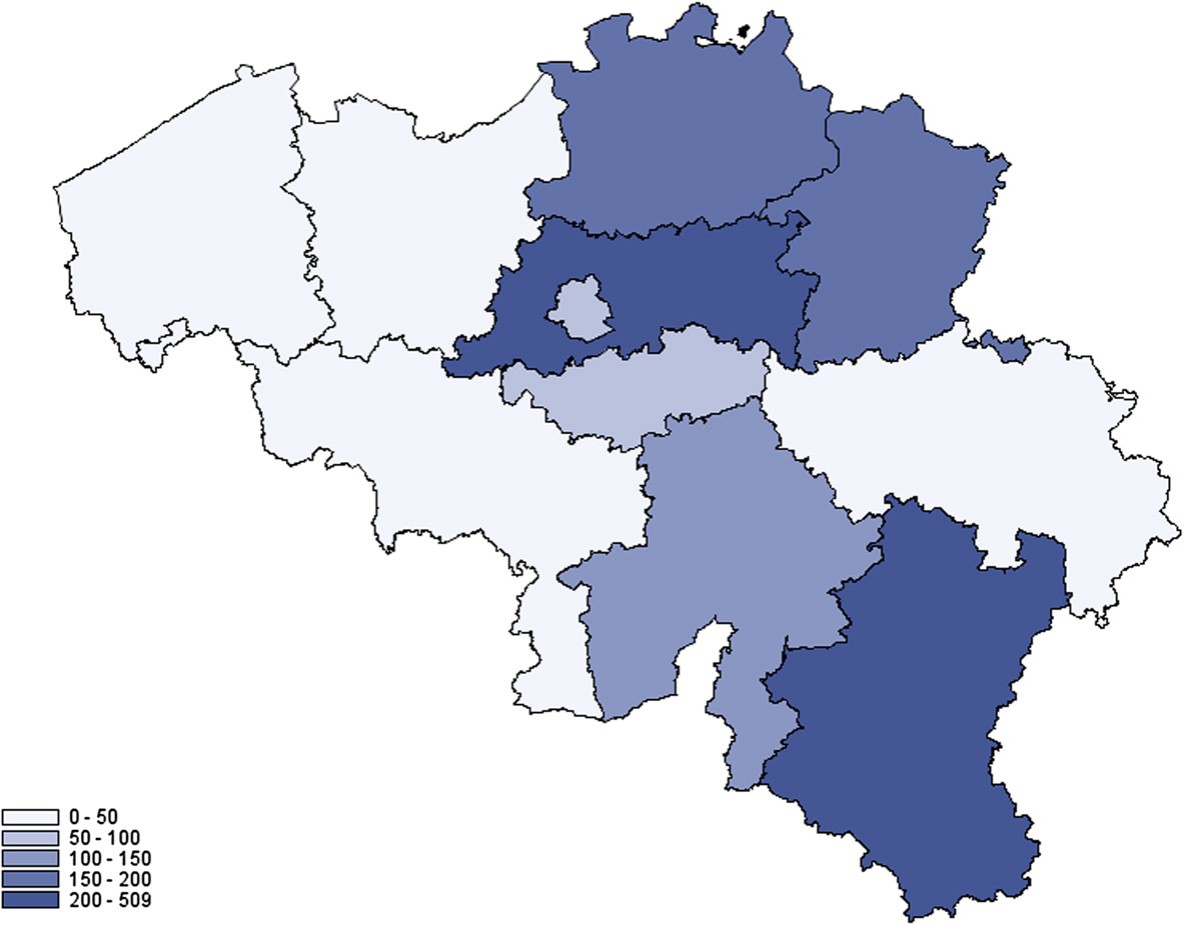
Reported cumulative incidence of positive laboratory tests for Lyme borreliosis by 100 000 inhabitants, by province, 2003–2012, Belgium

The distribution of the number of hospitalizations by province identifies Luxemburg, Limburg and Antwerp as the provinces reporting more hospitalizations for Lyme borreliosis (Fig. 5). The cumulative incidence for the period 2003 to 2010 was 228.8 hospitalizations/100 000 inhabitants for Luxemburg, 221.1 for Limburg and 153.5 for Antwerp. No significant changes were observed in the number of hospitalizations between the 2003–2006 and 2007–2010 periods (p = 0.78), and between the 2007–2008 and 2009–2010 periods (p = 0.94). In proportion to their respective population, the most positive tests for Lyme borreliosis reported by the SLN were for patients from the provinces Luxemburg, Flemish Brabant and Antwerp (Fig. 6). The respective reported cumulative incidences/100 000 inhabitants for the period 2003–2012 was 508.5 for Luxemburg, 301.2 for Flemish Brabant and 172.0 for Antwerp. A Wilcoxon test applied to all reported cumulative incidences by province neither showed significant changes between the 2003–2007 and 2008–2012 periods (p = 0.96), nor between the 2009–2010 and 2011–2012 periods (p = 0.75).

Men were slightly more likely to be hospitalized than women (58 %) and to have a positive serology result (52 %). Most hospitalized patients were either 5–9 years old children, or 45–69 years old adults, similar to the age groups presenting the most positive laboratory tests (5 to 14 years old children and adults aged 50 to 69 years). The age and gender distribution are comparable over the time period (data not shown).

## Discussion

Lyme disease is becoming a “hot topic” in Western countries. In Europe, an increase in Lyme borreliosis has been described in some countries, but not in others [5]. This is also the case in the countries neighboring Belgium. The Netherlands report an increase in consultations and hospital admissions for Lyme borreliosis between 1994 and 2009 [9, 10]. Eastern Germany, on the other hand, observes a decrease in Lyme borreliosis between 2009 and 2012 [13]. French reports show no increase in hospitalizations between 2004 and 2009, and also a stability in yearly Lyme borreliosis incidence rate (data from the general practitioners sentinel network) between 2009 and 2012 [14]. Switzerland describes a stable reported incidence between 2008 and 2011 [15]. However, comparisons within and between countries need to be looked at with caution, as surveillance methodology varies by country and other factors might explain some variations. For example in Belgium, a gradual increase in the number of Lyme borreliosis positive tests was observed during the beginning of the nineties, following the introduction of ELISA test reimbursement.

In Belgium, surveillance of Lyme disease occurs through a sentinel network of laboratories (SNL) reporting the number of positive laboratory tests since 1987 [21]. The reliability of the network is based on a stable participation of laboratories, covering around 50 % of all Lyme borreliosis tests carried out in Belgium (Berger N., unpublished observations), with weekly automatic extraction of data for more than 70 % of participating laboratories. The network allows to follow trends in incidence of laboratory confirmed tests, but this is only a partial picture of the incidence of the disease. In the context of increasing incidence of Lyme borreliosis in some European countries, the routine surveillance data were validated with information collected through the registration of minimum clinical (RMC) data from the hospitals.

Both data sources converge to the same result: we do not observe any increase in Lyme borreliosis in Belgium during the study period. However, early clinical manifestations of Lyme diseases, such as erythema migrans, are not covered by these surveillance systems, since laboratory testing is not recommended in an early stage and those manifestations do not require hospitalization. Yet, results of two studies carried out by the Belgian sentinel network of general practitioners did not show a significant increase of the incidence rate of tick bites and erythema migrans between 2003 and 2009 [17]. The incidence of erythema migrans per 10,000 patients in 2003–2004 and in 2008–2009 was 8.32 and 9.02 respectively (p > 0.05). Our SLN and RMC observations are congruent with those clinical results.

Despite a global stable trend during the 2003–2010/2003–2012 period, RMC and SLN data show yearly fluctuations. Climate changes, with higher temperatures during winter months and increased humidity, or the presence of snow, impact on tick abundance and can explain some of these yearly variations [22, 23]. Further studies are needed to explore the influence of climate on Lyme disease in Belgium. The observed difference in fluctuations over time between the SLN and RMC data could be due to the fact that part of the hospitalizations are due to late manifestations of Lyme borreliosis, which may occur years after the tick bite. As RMC only provides the diagnosis without any precision regarding the type of complication, we lack information to confirm or invalidate this hypothesis.

Other factors than climate can also impact on Lyme disease incidence. The number of positive laboratory tests depends on the prescription practice of physicians, which is influenced by the reimbursement of tests, the physician’s awareness of the disease, and individual insistence to be tested. The latters are both influenced by various factors, including media. However, during the study period, the positivity rate of tests carried out by the SNL remained stable (1.5 to 3.5 % of total tests) (WIV-ISP, unpublished data), and no statistical difference was observed in incidence of hospitalization and positive laboratory results between the different time periods.

The monthly distribution of RMC and SLN cases follows a seasonal pattern, with a very small number of cases during the winter, a gradual increase during the spring and summer periods, and a maximum peak during the end of the summer and the autumn. This is congruent with Ixodes ricinus phenology [23]. The number of nymphs collected in Belgium in 2009 and 2010 increased gradually during the spring, to reach a maximum in July (in 2009) and in June (in 2010) [23]. The peak of the number of hospitalizations and positive laboratory results is reached two to three months later, which corresponds to the time of onset of the first complications after a tick bite (early disseminated Lyme borreliosis).

The highest cumulative incidence of hospitalizations, and the highest incidence of inhabitants with positive laboratory tests during the study period are registered in the province of Luxemburg. This province has different geographical and climate characteristics compared to other Belgian provinces that can influence on tick density, with a higher proportion of forests, a higher altitude (around 500 meters versus sea level) and a wetter and colder climate, with snow during the winter. Other provinces that are most affected by Lyme borreliosis are Limburg, Antwerp and Flemish Brabant. These provinces comprise small field and forest areas, alternating with high densely populated urban areas, which can also impact on tick density [22, 23]. Although the geographical distribution presented here is based on the place of residence of the patient, which is not necessarily the place of infection, the distribution in our study is in line with reported results of tick collection [23]. Indeed in 2009 and 2010, high nymph densities were reported in the provinces of Flemish Brabant (49.0 to 59.4 nymphs/100 m^2^), Limburg (31.4 to 56.6 nymphs/100 m^2^) and Luxemburg (12.1 to 17.2 nymphs/100 m^2^).

The individual characteristics of RMC and SLN cases globally match the literature. A higher incidence in men than in women has previously been described [17], and could be due to a higher occupational risk, and also be related to certain leisure activities. Regarding the age distribution, the literature describes two groups mainly affected: children from 5 to 14 years, and adults from 50 to 64 years [5], as observed in our study.

Although the two data sources used here report comparable results, they both have strengths and limitations. The RMC database is based on compulsorily registration of all Lyme borreliosis hospitalizations from every general hospital in Belgium and is therefore expected to be exhaustive. Its main limitations are the delay needed to get the data, and a lack of precision regarding the symptoms, the ICD 9 code making no distinction between clinical manifestations. Moreover, since the data collected represent both principal and secondary diagnosis from all hospitalizations, the hospitalization incidences presented in this study do not reflect the severity of Lyme disease in Belgium but are rather used to appraise its trends.

The sentinel laboratory network is considered to be stable, and exists since more than 30 years. It nevertheless comprises several limitations. Firstly, if the network is representative at national level, its geographical repartition at provincial level is uneven. For example, the coverage of East Flanders and Flemish Brabant is high (between 80 and 90 %), whereas Namur, Liege and Limburg have a lower coverage (below 50 %). The reported incidence may thus be underestimated in the provinces with a lower coverage. Secondly, it gives a partial picture of the incidence, as laboratory tests are not recommended for patients presenting with an erythema migrans. Thirdly, a window period of undetectable antibodies should be considered if the blood analysis occurs within the first three weeks after the tick bite. Fourthly, laboratory tests only confirm the presence of anti-Borrelia antibodies, which does not necessarily means that the patient is suffering from Lyme disease: it could also be due to a previous symptomatic or asymptomatic Borrelia infection. Fifthly, the database may content some duplicates, as a patient is considered to be eligible for reinfection after a one-year period. However, as the database is repetitively used with the same parameters, this phenomenon should not have any impact on its global trend, which is of interest and regularly followed in public health.

Finally, we must note that routine SLN monitoring shows a new peak in positive tests for the year 2013, but the positivity rate remains stable. Further follow-up and surveillance remains essential.

## Conclusion

In Belgium, the surveillance of Lyme borreliosis is performed through three complementary sources: the WIV-ISP sentinel laboratory network, hospitalization data and a sentinel network of general practitioners. Those three sources indicate that there is no increasing trend in Lyme borreliosis during the 2003-2010/2012 period in Belgium. As Lyme borreliosis is a complex disease, the use of various data sources has to be maintained in the future, in order to monitor the epidemiological evolution of the disease and detect possible changes in trends. Furthermore, surveillance of the vector in Belgium should be strengthened. A new study involving the sentinel network of general practitioners will start in 2015, to follow-up on the incidence of tick bites and erythema migrans. The automatic extraction of data from a unique medical file in ambulatory medicine could facilitate clinical surveillance of Lyme disease in future.

## Abbreviations

RMC: Registration of minimal clinical data
SLN: Eentinel laboratory network
WIV-ISP: Belgian Scientific Institute of Public Health
